# Just a simple observational follow-up study?

**DOI:** 10.1186/1745-6215-16-S2-P90

**Published:** 2015-11-16

**Authors:** Lucy Culliford, Tracy Harris, Vhris Rogers, Usha Chakravarthy, Barney Reeves

**Affiliations:** 1 University of Bristol, Bristol, UK; 2 Queen's University, Belfast, UK

## 

Observational cohort studies are usually assumed to be simpler to set up and deliver than a RCT. But what happens when the cohort previously took part in a randomised CTIMP? Its anything but simple. We describe our experience of setting up an observational cohort study of patients who took part in the IVAN trial.

IVAN was a multi-centre RCT comparing bevacizumab with the standard treatment of ranibizumab for the treatment of wet age-related macular degeneration. Participants exited the trial on average 5 years ago, and if they continued to be treated under the NHS would have received ranibizumab. For this follow-up study no treatment is planned, data will be collected on treatment since the end of the trial, most recent visual acuity and imaging of the eye. Visual acuity and imaging data will be collected at a dedicated research visit if participants are willing to attend or from routine sources.

Challenges include

1. MHRA consider this follow-up study falls under the remit of the EU Clinical Trials Directive; i.e. that is a CTIMP, requiring amongst other things SAE reporting.

2. The trial population were elderly; a large proportion is likely to be too infirm to attend or have died. They may also have moved? How do we maximise the number of participants included to make sure the results are meaningful?

3. Some sites had low recruitment so the resources required to initiate and reactivate the site may not make their involvement worthwhile.

We will describe strategies we are using to overcome these challenges.

